# Reply: Irinotecan in patients with relapsed or cisplatin-refractory germ cell cancer: a phase II study of the German Testicular Cancer Study Group

**DOI:** 10.1038/sj.bjc.6601177

**Published:** 2003-09-09

**Authors:** C Kollmannsberger, C Bokemeyer

**Affiliations:** 1 Department of Hematology/Oncology/Immunology/Rheumatology, University of Tuebingen, Otfried-Mueller-Str. 10, 72076 Tuebingen, Germany

**Sir**,

In their letter, Powles *et al* disagree with our conclusions drawn from the results of a phase II study on single-agent irinotecan in patients with refractory germ cell cancer (Kollmannsberger *et al*, 2002b). At the same time, they question whether single-agent phase II studies may be the best way to identify new potentially active drugs in these patients. This statement is based on preclinical as well as on preliminary clinical data, which must, however, be thoroughly discussed.

Preclinical studies demonstrating an increased level of expression of topoisomerase I in germ cell cancer specimens or an impressive activity of SN 38, the active metabolite of irinotecan, in cisplatin-resistant germ cell cancer cell lines, form a strong rationale for the evaluation of irinotecan in refractory germ cell cancer patients. However, since we know from many other agents that an *in vitro* activity may not always translate into clinical activity, these preclinical findings do not allow to draw the conclusion that irinotecan is active in refractory germ cell cancer patients. Evidence for clinical activity of new agents can only be provided by prospective clinical trials. Single-agent studies are typically employed to obtain hints of the potential activity of a new agent and to form a rationale for potential combinations. Of course, single-agent studies cannot detect a synergism with other drugs. Combination regimens in patients with refractory germ cell cancer may lead to improved results, but these combinations should include active drugs. Our study investigating irinotecan in refractory patients included 15 patients and none of them responded. Thus, the probability for a false rejection of an active drug was less than 5% (study design according to Gehan, 1961), which makes a clinical activity with >20% response rate of irinotecan unlikely. We agree with Powles *et al* that the negative results of a single phase II study should still be considered carefully. However, our study investigating irinotecan single-agent therapy in patients with refractory germ cell cancer was the second study that tested a topoisomerase I inhibitor in these patients. Topotecan, the second clinically available topoisomerase I inhibitor, has also been ineffective in relapsed or refractory germ cell cancer patients (Puc *et al*, 1995).

Based on the proven, but moderate activity of single-agents such as paclitaxel, oral etoposide, oxaliplatin or gemcitabine with response rates of 15–20% and median overall survival periods of 3–6 months in patients with refractory disease, the investigation of combinations of active drugs is appropriate. Hinton *et al* (2001) were the first who combined two drugs and demonstrated the feasibility and efficacy of combination chemotherapy even in heavily pretreated germ cell cancer patients. The rationale for the combination of paclitaxel and gemcitabine, however, was based on the activity of both drugs when given as single-agent therapy (Motzer *et al*, 1994; Bokemeyer *et al*, 1996, 1999; Einhorn *et al*, 1999). In their letter, Powles *et al* report a very high response rate of 67% and a median survival of 11+ months for the combination of oxaliplatin, paclitaxel and irinotecan. These favourable results, although still do not prove the activity of irinotecan in these patients since the two other drugs, oxaliplatin and paclitaxel, have been shown to be active in refractory germ cell cancer (Bokemeyer *et al*, 1996; Kollmannsberger *et al*, 2002a). A high activity of oxaliplatin/paclitaxel has already been demonstrated in cisplatin-resistant patients with metastatic ovarian cancer indicating that this combination may act synergistically and can overcome cisplatin resistance (Faivre *et al*, 1999). Thus, although not yet proven in a formal study, the combination of the two agents is very likely to be also active in refractory patients. Thus, the results reported by Powles may have been achieved solely with oxaliplatin/paclitaxel and the potential additional benefit of irinotecan cannot be derived from that study. In addition, patient selection plays an important role. In the study by Miki *et al* investigating the combination of irinotecan and cisplatin in relapsed germ cell cancer patients, only four of 18 patients had previously received high-dose chemotherapy plus blood stem cell support and in three patients, cisplatin/irinotecan was used as second-line therapy indicating that this may have been a rather favourable patient group. Moreover, no details were given about the cisplatin sensitivity of these patients (Miki *et al*, 2002). Similarly in the ongoing study by Powles using a combination of oxaliplatin, paclitaxel and irinotecan, only 22% of patients were cisplatin-refractory and 22% had previously received high-dose chemotherapy, which demonstrates still a more favourable risk profile of these patients in contrast to our study. In all our previous studies on refractory patients, the majority of these patients had been pretreated with high-dose chemotherapy or were truly cisplatin-refractory.

Thus, despite a strong rationale from *in vitro* results that had led us to investigate the use of irinotecan in relapsed or cisplatin-refractory germ cell cancer, no evidence for a clinically meaningful activity of irinotecan has thus far been provided. We clearly agree that patients with refractory germ cell cancer need to be treated within prospective clinical trails in order to learn more about possible therapeutic strategies.

## References

[bib1] Bokemeyer C, Beyer J, Metzner B, Rüther U, Harstrick A, Weißbach L, Köhrmann U, Verbeek W, Schmoll HJ (1996) Phase II study of paclitaxel in patients with relapsed or cisplatin-refractory testicular cancer. Ann Oncol 7: 31–34 10.1093/oxfordjournals.annonc.a010473 9081388

[bib2] Bokemeyer C, Gerl A, Schöffski P, Harstrick A, Niederle N, Beyer J, Casper J, Schmoll HJ, Kanz L (1999) Gemcitabine in patients with relapsed or cisplatin-refractory testicular cancer. J Clin Oncol 17: 512–516 10080593 10.1200/JCO.1999.17.2.512

[bib3] Einhorn LH, Stender MJ, Williams SD (1999) Phase II trial of gemcitabine in refractory germ cell tumors. J Clin Oncol 17: 509–511 10080592 10.1200/JCO.1999.17.2.509

[bib4] Faivre S, Kalla S, Cvitkovic E, Bourdon O, Hauteville D, Dourte LM, Bensmaine MA, Itzhaki M, Marty M, Extra JM (1999) Oxaliplatin and paclitaxel combination in patients with platinum-pretreated ovarian carcinoma: an investigator-originated compassionate-use experience. Ann Oncol 10: 1125–1128 10572615 10.1023/a:1008334215414

[bib5] Gehan E (1961) The determination of the number of patients required in a preliminary and a follow-up trial of a new chemotherapeutic agent. J Chron Dis 13: 346–353 13704181 10.1016/0021-9681(61)90060-1

[bib6] Hinton S, Catalano PJ, Einhorn L, Kuzel T, Vaughn DJ, Wilding G (2001) Phase II trial of paclitaxel and gemcitabine in refractory germ cell tumors. J Clin Oncol 20: 1859–1863 10.1200/JCO.2002.07.158 11919245

[bib7] Kollmannsberger C, Rick O, Derigs H-G, Schleucher N, Schoeffski P, Beyer J, Schoch R, Sayer HG, Gerl A, Kuczyk M, Spott C, Kanz L, Bokemeyer C (2002a) Activity of oxaliplatin in patients with relapsed or cisplatin-refractory germ cell cancer: a study of the German Testicular Cancer Study Group. J Clin Oncol 20: 2031–2037 11956262 10.1200/JCO.2002.08.050

[bib8] Kollmannsberger C, Rick O, Klaproth H, Kubin T, Sayer HG, Hentrich M, Welslau M, Mayer F, Spott C, Kanz L, Bokemeyer C (2002b) Irinotecan in patients with relapsed or cisplatin-refractory germ cell cancer: a study of the German Testicular Cancer Study Group. Br J Cancer 87: 729–732 12232755 10.1038/sj.bjc.66000524 PMC2364262

[bib9] Miki T, Mizutani Y, Nonomura N, Nomoto T, Nakao M, Saiki S, Kotake T, Okuyama A (2002) Irinotecan plus cisplatin has substantial antitumor effect as salvage chemotherapy against germ cell tumors. Cancer 95: 1879–1885 12404281 10.1002/cncr.10918

[bib10] Motzer RJ, Bajorin D, Schwartz LH, Hutter HS, Bosl GJ, Scher H, Lyn P, Fischer P (1994) Phase II trial of paclitaxel shows antitumor activity in patients with previously treated germ cell tumors. J Clin Oncol 12: 2277–2283 7525885 10.1200/JCO.1994.12.11.2277

[bib11] Puc HS, Bajorin D, Bosl GJ, Amsterdam A, Motzer RJ (1995) Phase II trial of topotecan in patients with cisplatin-refractory germ cell tumors. Invest New Drugs 13: 163–165 8617580 10.1007/BF00872866

